# Responses of the *Allium cepa* L. to Heavy Metals from Contaminated Soil

**DOI:** 10.3390/plants13202913

**Published:** 2024-10-17

**Authors:** Ocsana Opriș, Ildiko Lung, Katalin Gméling, Adina Stegarescu, Noémi Buczkó, Otilia Culicov, Maria-Loredana Soran

**Affiliations:** 1 National Institute for Research and Development of Isotopic and Molecular Technologies, 67-103 Donat, 400293 Cluj-Napoca, Romania; ocsana.opris@itim-cj.ro (O.O.); ildiko.lung@itim-cj.ro (I.L.); adina.stegarescu@itim-cj.ro (A.S.); 2 Centre for Energy Research, 29-33 Konkoly-Thege Miklós, 1121 Budapest, Hungary; gmeling.katalin@ek-cer.hu (K.G.); buczko.noemi@ek-cer.hu (N.B.); 3 Joint Institute for Nuclear Research, 6 Joliot-Curie, 141980 Dubna, Russia; culicov@nf.jinr.ru; 4 National Institute for Research and Development in Electrical Engineering ICPE-CA, 313 Splaiul Unirii, 030138 Bucharest, Romania

**Keywords:** *Allium cepa* L., heavy metals, assimilatory pigments, polyphenols, antioxidant activity, elemental content

## Abstract

Heavy metals can accumulate and migrate in soil environments and can negatively affect crops and consumers. Because an increased incidence of chronic diseases can be observed, food security has become a high-priority concern. In the present work, we evaluate the impact of heavy metals on bioactive compounds and elemental content from onions. Plants were grown in the absence and presence of various concentrations of heavy metal salts (Pb, Mn, Cu, Zn, Ni and Cd). The influence of heavy metal salts on onions was evaluated by analyzing the content of bioactive compounds, antioxidant capacity, and elemental content. The variation of assimilatory pigments, total polyphenols content, and antioxidant capacity increased or decreased depending on the heavy metal added to the soil as well as on the amount added. Regarding the amount of bioactive compounds, it increased between 6.79 and 34.39% or decreased between 4.68 and 62.42%. The content of ten elements in plants was reported, as well as elemental mutual correlation and correlation of element content with biologically active compounds and antioxidant capacity.

## 1. Introduction

Rapid industrialization has become an important problem during the last year for the entire world because this industrialization destroys the balance of the whole ecosystem by heavy metal contamination of our soil and groundwater, especially with lead (Pb), cadmium (Cd) and copper (Cu). The heavy metals contaminate crops and vegetables as they reach the groundwater and humans are at risk of important adverse health effects due to the consumption of vegetables that are grown in contaminated soils. Heavy metals such as Hg, Sn, Pb, As, and Cd can cause damage to human health even in small quantities. In the last few years, the related risks have been attracting wide attention from different sectors and investigators all over the world. Also, it is very important to analyze and to understand the pattern of heavy metals in the soil and identify the sources of these metals to determine the biological risk [1,2].

Heavy metals contamination is insidious, long-lasting and, in most cases, irreversible. On the other hand, the heavy metals that have migrated into the soil cannot be decomposed and thus remain in the soil for a long time and, having a long biological half-life, can accumulate and harm the environment with consequences for human health by accumulation in various organs of the body, which leads to undesirable consequences [3,4,5].

Onions (*Allium cepa* L.) are among the most commonly and oldest vegetables produced and consumed, with an estimated production in a year of more than 90 million tons worldwide [6]. It is known that the onion accumulates a high concentration of lead (Pb) and cadmium (Cd), while, according to the literature, growth inhibition at higher concentrations of copper (Cu) appears [7,8]. In 2013, Gebrekidan et al. published a study about the concentrations of heavy metals found in irrigated soil samples obtained from Tahtay Wukro, Ethiopia, with worrying results (overall results of soil samples ranged from 2.62–827, 1.4–51.6, 25.5–33.6, 23.5–28.2, 2.52–25.1,15–17.8,3–4, 2.5–40.49 and 0.7–0.8 mg/kg for Mn, Zn, Cr, Ni, Cu, Co, Pb, Fe and Cd, respectively) [9]. They also found that Pb accumulates the most in vegetable samples. Green peppers and lettuce accumulate high amounts of Cu and Zn; excessive amounts of Fe, Mn, Cr, Cd, Ni and Co have been found in Swiss chard crops, while in the case of lettuce and tomatoes, higher amounts of Cd were found. Green peppers, tomatoes and onions presented a higher concentration of Pb. Consumption of Cd- and Pb-contaminated vegetables when their concentration is higher than the permissible limits may cause human health hazards [10,11,12].

The fertilizers and pesticides, very intensively used in agriculture, also contribute greatly to the pollution of land and agricultural products. Purbalisa et al. found an important quantity of lead in agricultural land and they propose to reduce it by using a chelating agent [13]. In their study, the onion (Bima Kurut variety) was used and they followed the growth and yield of shallots, and the metal content of ground lead before and after the application of chelating agents to the soil and plants. The percentage of lead in the soil is reduced by more than 50% after treatment with a chelating agent [13].

Onions, which are a bulbous vegetal, react well to the addition of organic manures and this kind of fertilizer has improved the composition of compounds found in onions (such as polyphenols, flavonoids, antioxidants, carbohydrates and sugar) [14,15]. Petrovic and Pokluda, in 2020, proved the positive influence of organic fertilizers on vitamin C, antioxidants and carotenoids under controlled conditions [16]. The application of poultry manure as an organic fertilizer is essential for improving soil productivity and crop production, but feed additives containing PTEs (potentially toxic elements) are intensively applied in the poultry industry and generate several problems, including the accumulation of PTEs in plants associated with health risks in humans [17]. The concentrations of PTEs have been found to be less than the permissible levels determined by the U.S. EPA (1994) (Cu: *p* < 0.05 and Cr: *p* < 0.01); the concentrations of Pb within all food plants have been significantly (*p* < 0.01) higher and over the permissible limits determined by FAO/WHO (2001). The levels of Cd, Cu and Pb have been higher than 1 for wheat, barley, radish and onion, suggesting that the consumers would be significantly exposed to risk (HRI > 1) [17,18,19].

Shokri et al. assessed the impact of heavy metals on humans consuming onions by testing 22 samples of onion (e.g., red, yellow, and white varieties) randomly sampled from different regions of Kurdistan, Hamedan and Kermanshah during two different seasons. According to the results, two heavy metals, Pb and Cd, were found in almost all onion samples collected from different areas, and their level exceeded the allowable range recommended by the standard [8,20].

Recently, Li et al. assessed the contamination and risk of heavy metals in crops or farmland near mines. They determined the content of heavy metals such as Cr, Ni, Cu, As, Cd, Pb and Zn in some soil sampling sites of agricultural land and for some vegetables (including onions) [21]. They concluded that the heavy metals could be released into the environment through atmospheric fallout, surface water pollution (including acid mine runoff) or groundwater seepage, which poses serious environmental risks. It was established that agricultural land contains Cd exceeding the regulatory values to a greater extent than other metals, and Zn showed the highest abundance and accumulation with the most obvious contamination (maximum concentration was 2382.207 mg/kg). In the case of As, Cd, Pb and Zn, the average concentrations were 2.927, 91.216, 9.144 and 15.346 times higher than the background values of the soil environment in Niujiaotang and, respectively, 1.805, 31.787, 5.980 and 17.390 times higher than the background values of the soil environment of Guizhou province. Heavy metal contamination of crops decreases in the following way: Chrysanthemum ˂ coronarium ˂ radish rice ˂ spring onion ˂ Chinese cabbage ˂ pak choi, and the individual heavy metal contamination of crops follows the order Zn > As > Cr > Cd > Ni > Pb and it was highlighted that the farmlands were severely polluted [2,21,22].

Onions are among the most important crops and their cultivation and production are continuously increasing [23]. Onions contain many phytomolecules, such as polyphenols, phenolic acids, flavonoids, ascorbic acid, and sulfur compounds [24]. According to various studies, onions have many health benefits, such as anti-diabetic, hypotensive and lipid-lowering properties. [24,25,26]. In this study, the onion was chosen because the abiotic stresses led to the retardation of onion growth and the development of the bulb as well as production losses [27].

The goal of the present study was to investigate the influence of six heavy metals (Cd, Cu, Zn, Mn, Ni and Pb) on biologically active compounds and on the element content of onions (*Allium cepa* L.). More specifically, three different concentrations of the studied heavy metals were selected to assess their impact on chlorophyll *a*, chlorophyll *b*, carotenoids, polyphenols, antioxidant capacity, and the variation of the multielement content.

## 2. Results and Discussion

### 2.1. Onion Extracts Analysis

#### 2.1.1. Assimilatory Pigments

**Figure 1:**
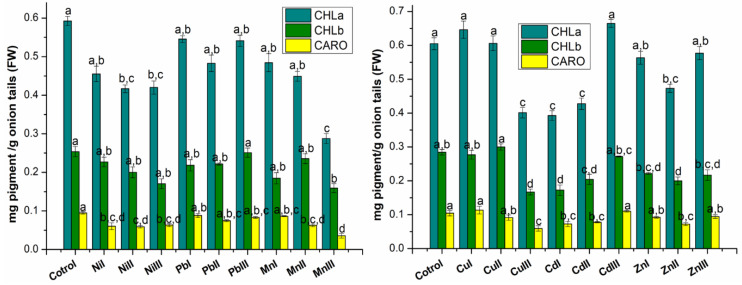
The assimilatory pigments (chlorophyll *a*—CHL*a*; chlorophyll *b*—CHL*b*; carotenoids–CARO) content expressed as mg pigment/g fresh weight (FW) of *Allium cepa* L. (onion tails) extracts grown in the presence of heavy metals (Ni, Pb, Mn, Cu, Cd and Zn) at different concentrations compared to control onions. I—concentration under accepted limit, II—maximum accepted limit and III—above maximum accepted limit. These concentrations correspond as follows: Ni (15, 30, 75 mg kg^−1^); Pb (15, 30, 50 mg kg^−1^); Mn (675, 1350, 1500 mg kg^−1^); Cu (15, 30, 100 mg kg^−1^); Cd (0.75, 1.5, 3 mg kg^−1^); Zn (75, 150, 300 mg kg^−1^), a, b, c, d—the statistically considerable differences among groups (*p* < 0.05).

The variation of assimilatory pigments in onions grown in the presence of heavy metals at different concentrations compared to control onions is presented in Figure 1.

In all the plants grown in the presence of heavy metals (Ni, Pb and Mn), the amount of pigments decreased compared to the control. The greatest decrease was registered in the case of those grown in the presence of Mn at a concentration above the maximum accepted limit. The decreases vary from 7.90–51.46% in the case of chlorophyll *a*, 7.18–37.18% in the case of chlorophyll *b* and 7.26–62.42% in the case of total carotenoids.

The variation of pigment content in the case of onions grown in the presence of Cu, Cd and Zn metals compared to the control, depends on the type of pigment and the type and concentration of the metal. Thus, in the case of chlorophyll *a*, its amount increased in the case of Cu I (6.79%), Cu II (insignificant) and Cd III (9.83%) plants. In the case of the other plants, the amount of chlorophyll decreased from 4.68–35.06%. The greatest decrease was recorded for Cd I. In the case of chlorophyll *b*, only in the case of Cu II plants was there an increase of 5.52% compared to the control. In the case of all other plants, the amount of chlorophyll *b* decreased from 2.6–41.30%. The greatest decrease was obtained for Cu III. Comparing the content of carotenoids, it was observed that it varies very little compared to the control, except for Cu III, Cd I, Cd II and Zn II. For the latter ones, a decrease in carotenoid content was obtained compared to the control by 43.06%, 30.33%, 25.36%, and 30.91%, respectively.

#### 2.1.2. Total Polyphenols

**Figure 2:**
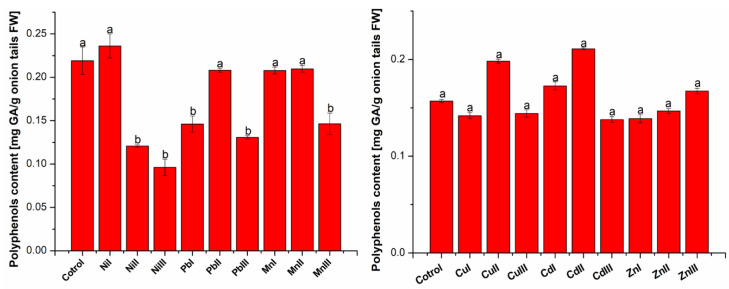
Total polyphenols content expressed as mg gallic acid (GA)/g fresh weight (FW) of *Allium cepa* L. (onion tails) extracts grown in the presence of heavy metals at different concentrations (I—concentration under accepted limit, II—maximum accepted limit and III—above maximum accepted limit) compared to control onions. The heavy metal concentrations correspond as follows: Ni (15, 30, 75 mg kg^−1^); Pb (15, 30, 50 mg kg^−1^); Mn (675, 1350, 1500 mg kg^−1^); Cu (15, 30, 100 mg kg^−1^); Cd (0.75, 1.5, 3 mg kg^−1^); Zn (75, 150, 300 mg kg^−1^), a, b—the statistically considerable differences among groups (*p* < 0.05).

The amount of total polyphenolic compounds (Figure 2), expressed as mg gallic acid (GA)/g fresh weight (FW) plant, was determined from the linear equation of the standard calibration curve: y = 0.5782x + 0.0063 (R^2^ = 0.9993).

It was observed that in the case of monitoring the influence of heavy metals (Ni, Pb and Mn) on the total polyphenols content extracted from onion tails, a higher value was obtained only for those grown in the presence of Ni at a concentration under the accepted limit. The increase was 7.71%. For all the other plants, the values were lower than the control. The decrease in total polyphenols content was in the range of 4.29–56.09%. The greatest decrease was registered in the case of plants exposed to Ni concentrations of the maximum accepted limit and at a concentration above the maximum accepted limit.

The amount of total polyphenols increased in Cu II, Cd I, Cd II and Zn III plants from 6.62–34.39%, compared to the control, and in the case of the others, it decreased from 6.50–12.33%. The greatest decrease was registered for Cd III.

#### 2.1.3. Antioxidant Capacity

**Figure 3:**
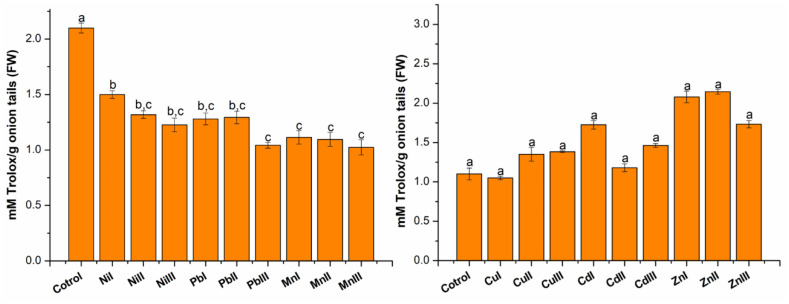
Antioxidant capacity expressed as mM Trolox/g fresh weight (FW) of *Allium cepa* L. (onion tails) extracts grown in the presence of heavy metals at different concentrations (I—concentration under accepted limit, II—maximum accepted limit and III—above maximum accepted limit) compared to control onions. The heavy metal concentrations correspond as follows: Ni (15, 30, 75 mg kg^−1^); Pb (15, 30, 50 mg kg^−1^); Mn (675, 1350, 1500 mg kg^−1^); Cu (15, 30, 100 mg kg^−1^); Cd (0.75, 1.5, 3 mg kg^−1^); Zn (75, 150, 300 mg kg^−1^), a, b, c—the statistically considerable differences among groups (*p* < 0.05).

The results for antioxidant capacity (Figure 3) expressed in mM Trolox equivalents (mM Trolox/g fresh weight (FW) of onion tails) were obtained using the linear equation of the standard calibration curve: y = 0.1792x + 0.0102 (R^2^ = 0.9991).

In the case of monitoring the influence of heavy metals (Ni, Pb and Mn) on the antioxidant capacity of onion tails, it was observed that at all three concentrations, lower results were obtained compared to the control plants. The decrease in antioxidant capacity was in the range of 28.58–51.22%. The greatest decrease was registered in the case of plants exposed to concentrations above the maximum accepted limit.

In the case of monitoring the influence of heavy metals (Cu, Cd and Zn) on the antioxidant capacity of onion tails, it was observed that at all three concentrations of the heavy metals, except for Cu at a concentration under the accepted limit where it decreased insignificantly, higher results were obtained compared to the control plants. The increase in antioxidant capacity was in the range of 7.21–95.00%. The greatest increase was registered in the case of plants exposed to Cd at a concentration under the accepted limit and Zn at all three concentrations.

### 2.2. Element Content in Plants

The onion and its main constituents in specific doses have proved plenty of advantages, including free radical scavenging and antioxidant properties, anticholesterolemic, anti-heavy metal toxicity and others [24] which can only be preserved if its bioactive compounds, antioxidant capacity and elemental content are not negatively affected during plant growth, which can occur in different environmental conditions, including in polluted soils. When studying the response of onions to various factors, researchers rarely pay attention to onion leaves, despite the fact that they are very widely used throughout the world. They usually focus on onion bulbs. The content of certain elements in the bulbs can be much lower than in the leaves [28]. Therefore, the use of the former for comparison is not very useful, but the absence of data for the leaves forces us to resort to them.

**Figure 4:**
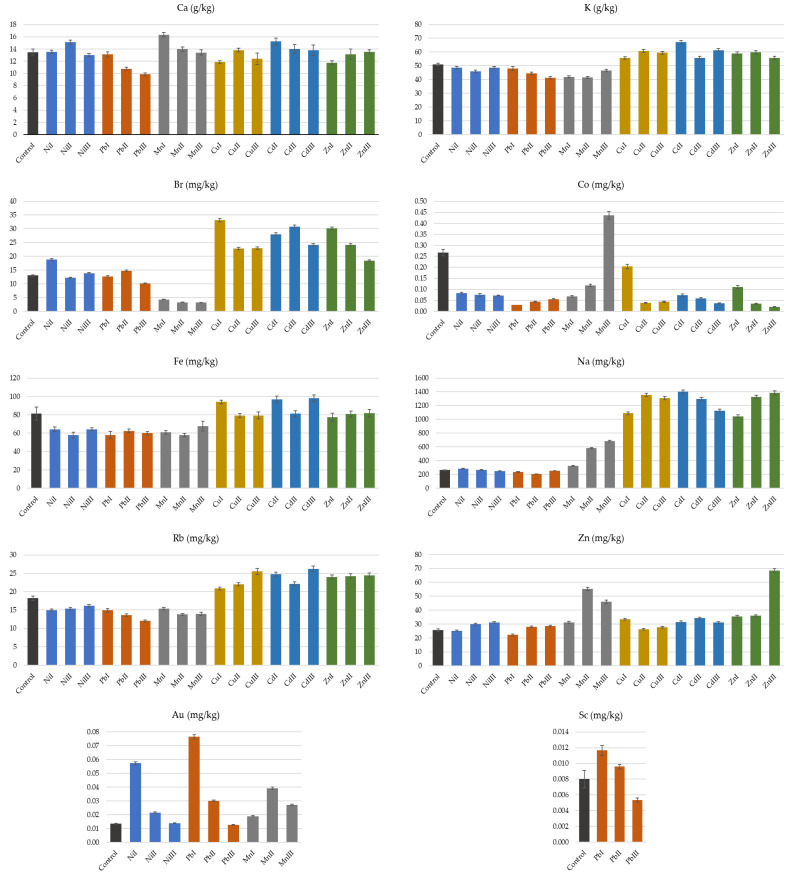
Comparative diagram of the element (Ca, K, Br, Co, Fe, Na, Rb, Zn and Au) content (mg/g) in *Allium cepa* L. (onion tails) grown in the presence of heavy metals at different concentrations (I—concentration under accepted limit, II—maximum accepted limit and III—above maximum accepted limit) compared to control onions. The heavy metal concentrations correspond as follows: Ni (15, 30, 75 mg kg^−1^); Pb (15, 30, 50 mg kg^−1^); Mn (675, 1350, 1500 mg kg^−1^); Cu (15, 30, 100 mg kg^−1^); Cd (0.75, 1.5, 3 mg kg^−1^); Zn (75, 150, 300 mg kg^−1^).

In our study, 14 chemical elements could be determined in the samples with the k0-INAA method after long irradiation. All results have quoted uncertainties for k = 1 standard deviation and include a 1.8% systematic uncertainty contribution. The interpretation is limited to 10 elements that could be determined in all the samples of an experimental setup, including the control ones. Br, Ca, Co, Fe, K, Na, Rb and Zn were determined in all experimental setups. Au was determined in setups with Ni, Pb and Mn while Sc was in setups with Pb only (Figure 4).

Among the macronutrients, Ca and K were determined. The Ca values obtained in our study varied between 9.87 and 16.32 g/kg and were about 15 times higher than those reported for various Polish and Czech onion bulb cultivars [29,30], respectively. The same sources reported much lower K values than those obtained in our study. Soil exposure to heavy metals induced relative increases of Ca compared to the control up to 21%, for the lowest Mn rates and a decrease up to 27% when the highest Pb rate was applied. None of the experimental settings showed a significant variation of the Ca for all the rates used. However, the gradual increase of Pb rates in the soil is strongly anticorrelated with the Ca content in the leaves (R^2^ = −0.96) (Table 1). Zinc applications to soil both as ZnSO_4_ and ZnNa_2_-EDTA tended to decrease leaf Ca [31].

Each of the rates of Ni, Pb and Mn used led to a significant decrease in K in plants compared to the control, but only for Pb, the decline was strongly anticorrelated with the rate increase. (R^2^ = −0.99). At the same time, the use of Cu, Cd and Zn induces a significant increase of K regardless of the rate of the heavy metal used, but the variation is not strongly correlated with the rate of the heavy metal (R^2^ < 0.62).

Among the micronutrients, Fe, Na and Zn were determined (Figure 4). Zn content ranged from 22.15 to 70 mg/kg and it is higher than the maximum value of 18.37 mg/kg reported for scallion grown in Thailand [32], being similar to values ranging from 31.75 up to 70.16 mg/kg reported in onion bulbs produced in Bangladesh [33]. Data reported on onion stem and leaf varied from 3.73 to 44.81 and 31.2 up to 92.23 mg/kg, respectively and do not exceed the limits fixed by FAO/WHO [19,34]. The values obtained in onions grown in the vicinity of a Korean Cu-W mine varied from 47.4 up to 256 mg/kg [35]. The zinc content of onion leaves correlated significantly with applied Zn rates from both ZnSO_4_ and ZnNa_2_-EDTA treatments [31]. In our study, the increase in the concentration of Ni, Mn and Zn in the soil correlated strongly with the variation of the Zn content in the plant. Although the Zn content in the plant increased significantly regardless of the Cd rate applied to the soil, variations were not strongly correlated. The addition of Pb to the soil first caused a decrease of Zn in plants and then a progressive increase in it.

Leaf Fe reacted similarly to K when Ni, Pb and Mn were applied. Plants lost from 17 to 29% of the control Fe, depending on the heavy metals rate applied to the soil. The use of Zn did not influence the plant Fe. Cu and Cd application tended to increase leaf Fe, but not for all rates. The concentration of Fe in onion tails varied from 57.9 up to 98 mg/mg, which is one order of magnitude lower than those reported by Bedassa et al. [28] and FAO/WHO [19] limits but higher than those reported for Polish onion bulbs [29]. Attempts to correct Cu deficiency in soil by means of CuSO_4_, Cu-EDTA and CuO NP [36] showed a variation of Fe in onion bulbs from 143.7 up to more than 150.7 mg/kg. Leaf Fe fell in the ranges 28.8–48.4 mg/kg and 43.1–48.4 mg/kg for ZnSO_4_ and ZnNa_2_-EDTA, respectively [31]. Zn decreased Fe content below that of the control except that Fe increased at the lowest ZnSO_4_ rate.

Plant Na content increased sharply regardless of the Mn, Cu, Cd and Zn rates added to the soil. The relative increase compared to the control reached 432% for Cd. The progressive increase of Mn and Zn rates in the soil also led to the progressive increase in plant Na in plants (R^2^ = 0.95 and R^2^ = 0.83, respectively) (Table 1). Soil treatment with ZnSO_4_ and ZnNa_2_-EDTA caused the opposite behavior of Na content in onion leaves [31]. With the first treatment, leaf Na decreased below that of the control but increased significantly with the second treatment. With Pb, leaf Na significantly decreased regardless of the rate, reaching up to 22% when 30 mg/kg were applied. The lowest Ni rate induced a significant increase of Na, while the other rates decreased leaf Na, the variation Ni in soil and Na in the plant being antagonistic. Bulb Na reported by Mlček varied from 56.65 up to 110.22 mg/kg and depended much on cultivar and seasonal variety [30]. Our experimental values were from 205 up to 1400 mg/kg closer to those reported for leaves after soil-applied Zn treatments [31].

Plant Br increased significantly at all Cu, Cd and Zn rates, but stronger at lower rates and decreased at higher rates. The gradual increase of the Mn rate is strongly correlated with the Br decrease (R^2^ = −0.9) (Table 1). No defined trends from Ni and Pb treatments were evident in the leaf concentration of Br.

Ahmad and Ansari reported values for onion bulbs from 0.16 up to 0.84 mg/kg [34]. Our results varied from 0.02 up to 0.44 mg/kg and were consistent with those reported onion leaves grown in the proximity of an iron-steel factory in Turkey of 0.081–0.428 mg/kg [37].

Plant Co decreased significantly regardless of the metal applied and rate, the variations in Co and metal used being antagonistic (R^2^ = −0.86–−0.65). The exception was Mn treatment, which first led to a significant decrease in the Co content and then to its increase. Since there are limited data on onion Co, the available information refers to a study analyzing different parts of three different colored onions but leaves [6] only. Current values lay within 0.02 and 0.44 mg/kg and exceeded the literature data of 0.0029–0.0661 mg/kg.

Ni, Pb and Mn applications tended to significantly decrease the plant Rb, the loss increasing along with the Pb and Mn treatment rate (R^2^ = −0.96 and −0.98, respectively). The variation of the leaf Rb positively correlated with the increase in the concentration of Cu, Cd and Zn in the soil (Table 1).

Ni and Pb application induced a huge increase of leaf Au at a minimum rate, but the rate increase led to a smaller increase in plant Au. At the maximum Pb rate, the leaf Au decreased below that of the control. Mn applied at all rates significantly increased Au content, the variation in plant Au correlating positively with the variation of the Mn rate in the soil.

As shown in several publications, under normal conditions, Sc cannot be actively taken up by plants [38,39]. Even though several reports show that Sc can be carcinogenic to plants [40] or have an inhibitory influence on mitosis and root elongation, as Al does [41], other studies have shown that, under certain conditions, scandium can have stimulatory effects on various vegetal varieties [42]. It was, therefore, decided to keep information about Sc in the paper, despite the fact that it could only be determined for the Pb treatment. A low Pb rate increased the Sc content in the plant, but the maximum Pb rate, on the contrary, decreased it. The progressive application of Pb decreased all determined elements in the plant except Zn, Au and Sc. The concentration of Zn first decreased at low levels of Pb in the soil but then increased significantly, while the concentration of Au and Sc increased particularly strongly at a minimum rate of Pb but decreased sharply with the increase of the concentration of heavy metals applied.

Co was released from the plant regardless of the treatment applied. Plant Br, K, Na, Rb and Zn concentration increased with applied Cu, Cd and Zn, the relative increase to control being over 297% for Na. With Mn, the plant significantly lost Br, Fe, K and Rb, but strongly assimilated Zn, Au and Na and some Ca, whose assimilation seems to be least affected by the additional presence of selected heavy metals in the soil. Increasing the Ni rate as well as the Mn rate, plant Co, Fe, K and Rb were diminished, but leaf Zn and Au increased.

### 2.3. Correlation of Element Content in Plants by Experimental Setup

With Ni, plant Ca and K variations correlated negatively as well as micronutrients Zn and Na. The decrease of K correlated with that of Fe, Co and Rb, while Au, Na and Br vary similarly (Table 2).

Treatment with Pb revealed several correlation groups. Plant Zn correlated negatively with the gradual decrease of Au, Sc and Ca content in plants. Rb, Ca, Co, Fe and K correlated well with each other, being depleted regardless of the Pb rate. Plant Au and Sc correlated well to each other, both increased at a low rate and significantly decreased at a high rate.

Mn application led to significant antagonism of Br with Na, Zn and Au on one side, Co and Ca on the other one and Rb with both Zn and Au on a third. Conversely, Br highly correlates with Fe, K and Rb. Plant Na forms a correlation cluster with Zn and Au.

Two clusters of positive correlations, Fe-Zn-Br and K-Na-Rb, were identified for Cu treatment. The first mentioned cluster is anticorrelated with Ca and the second one is anticorrelated with Co.

With Cd, plant Na, Br, Ca, K, Rb and Zn significantly correlated to each other, but negatively correlated with Co. Fe correlated with both K and Rb. Zn treatment led to an extraordinarily high correlation of Ca and Fe and a negative correlation of Co with the cluster of positive correlation Na-Rb-Br-K on one side and with Zn on the other.

The variation in Co content is always highly negatively correlated with the variation in K, Na, Rb and Zn when Cu, Cd and Zn are applied to the soil, apart from the Zn-Co correlation in the presence of Cu, which is close to zero.

### 2.4. Correlation of Element Content in Plants with Bioactive Compounds and Antioxidant Capacity by Experimental Setup

Table 3 shows the values of the correlation coefficients calculated between the content of each element in the plants and the bioactive compounds as well as the antioxidant capacity.

For the treatment with Ni, it may be noticed that the variation of photosynthetic pigments was associated with the variation of Co, Fe, K and Rb contents, while the variation of total polyphenol was also positively correlated with the variation of these elements, but with correlation coefficients under 0.56. On the other hand, the variation of total polyphenol content correlated with the variation of plant Na. The variation in the plant Zn is antagonistic to both the variation in the bioactive compounds and the antioxidant capacity, with minimum correlation values (R^2^ = −0.999) for the total polyphenol content.

The addition of Pb to the soil also led to positive correlations of antioxidant capacity with Co, Fe, K and Rb. Plant Ca variation is associated with this group of elements. The variation of the total carotenoid content correlated with the Ca-Co-K-Rb cluster with a maximum correlation coefficient of 0.86. It can be noticed that there were high correlation coefficients of photosynthetic pigments and plant Na. Antagonism was noted between the variation of plant Au and Sc and chlorophyll *b* only.

With Mn, plant bioactive compounds negatively correlated to Na variation in the plant as well as antioxidant capacity. The variation in antioxidant capacity went along with the variation in plant Fe, K and Rb content. Plant Rb correlated with chlorophyll *a* and carotenoid, too. At the same time, plant Zn behavior is antagonistic to those of antioxidant capacity and total content of carotenoids.

In the presence of additional Cu in the soil, the antioxidant capacity, K and Rb varied likewise, but opposite to the variation of Co and Fe. The variation of Rb content negatively correlated with the photosynthetic pigments. The total carotenoid content varied similarly to the plant Co content. A positive correlation was observed between Ca and the variation of total polyphenols.

Orsuamaez and his team reported that total polyphenol content responded negatively to cadmium-increased exposure and assimilation by immersing onion bulbs in Cd solutions of various concentrations [43]. A relevant negative correlation between the variation of Br and Co content and photosynthetic pigments with Cd was revealed. Variations in *chlorophyll b* and Na content negatively correlated, too. At the same time, variations in antioxidant capacity, Ca, Fe, K and Rb content positively correlated.

The treatment with Zn showed a very high positive correlation between the variation in Br, K, Na and Rb and antioxidant capacity, the latter one being negatively correlated to the variation in Co. Variations in K, Na and Rb content were antagonistic to the variation in photosynthetic pigments, the lowest values of coefficients being calculated for *chlorophyll b*.

The variation in K and Rb content was always highly correlated with the variation in antioxidant capacity, regardless of the treatment applied. If the additional presence of heavy metals in soil induced a decrease of K and Rb as treatments with Ni, Pb and Mn did, a decrease in antioxidant capacity was also noticed. On the contrary, a simultaneous increase of K, Rb content and antioxidant capacity occurred when Cu, Cd and Zn were applied to the soil.

## 3. Materials and Methods

### 3.1. Plant Growth Conditions

Chives (Agrosel, Câmpia Turzii, Romania) were sown at a depth of 1 cm in plastic pots (0.81 L, 13.5 cm Ø) containing 550 g of garden substrate with active humus and fertilizer for 6 weeks (Agro CS, Slovakia, 50 L). The garden substrate has the following physicochemical characteristics: pH = 5.5 ± 0.5, N-at least 0.1 m/m%, P_2_O_5_-at least 0.01 m/m%, and K_2_O-at least 0.03 m/m%. In this study, copper (II) chloride dihydrate, cadmium acetate dihydrate (Merck, Darmstadt, Germany), zinc acetate dihydrate, manganese (II) chloride tetrahydrate, nickel chloride, and lead (II) sulfate were selected with the following concentrations: I-one under accepted limit, II-maximum accepted limit and III-above maximum accepted limit. These concentrations correspond as follows: Cu (15, 30, 100 mg kg^−1^); Cd (0.75, 1.5, 3 mg kg^−1^); Zn (75, 150, 300 mg kg^−1^); Mn (675, 1350, 1500 mg kg^−1^); Ni (15, 30, 75 mg kg^−1^) and Pb (15, 30, 50 mg kg^−1^). During the first watering, the plants were watered with 200 mL of ultrapure water in which the salts of heavy metals were dissolved, then the watering was carried out every 4 days with 100 mL of ultrapure water. Also, control chives were sown only in a garden substrate. The pots were placed in a climate chamber (Memmert GmbH, Bavaria, Germany) under controlled light conditions (for 12 h from 24 h), 60% humidity, and a day/night temperature cycle of 20/10 °C. Three replicates of each treatment were grown and the plants were harvested at 6 weeks after sowing.

### 3.2. Onion Tails Analysis

#### 3.2.1. Determination of the Content of Assimilating Pigments

The assimilating pigments were extracted from the fresh green onion tails with acetone and followed the steps described by Soran et al. [44]. The extracts thus obtained were analyzed with a T80 UV-VIS spectrophotometer (PG Instruments Limited), recording absorbances in the range of 400–750 nm. The amount of the targeted assimilatory pigments (chlorophyll *a* and *b*, total carotenoids) was determined with the equations presented by Lung et al. [45].

#### 3.2.2. Determination of the Total Polyphenolic Content

The method of obtaining the hydroalcoholic extracts from onion tails and the determination of the content of total polyphenols was determined according to Soran et al. [44]. The extracts were obtained by grounding fresh onion tails (1 g) in the presence of liquid nitrogen and adding 15 mL solvent (60% ethanol) for 3 min after which the mixture was exposed to sonication for 30 min at room temperature. Further, the mixture was centrifuged at 7000 rpm for 10 min and the supernatant was decanted and stored in the refrigerator at 4 °C prior to analysis. All extracts were obtained in triplicate. The content of total polyphenols was determined by the Folin–Ciocalteu method. Thus, 1 mL of extract and 0.5 mL of Folin–Ciocâlteu reagent was added to a 10 mL volumetric flask containing 5 mL of double distilled water. The content of the flask was mixed and after 3 min of standing, 1.5 mL of Na_2_CO_3_ (5 g L^−1^) was added and the volume of the flask was adjusted with double distilled water. The samples were placed in a water bath at 50 °C and kept for 16 min, after which they were removed and allowed to cool to room temperature. The absorbances of the samples were read in relation to the double distilled water at 765 nm. To determine the total content of polyphenols, a calibration curve was drawn using as the standard a gallic acid (GA) solution in the range of 0.001–0.800 mg mL^−1^.

#### 3.2.3. Determination of Antioxidant Capacity

The antioxidant capacity of the hydroalcoholic onion tail extracts was determined following the method of Brand-Williams et al. [46], slightly modified. The working procedure is the one specified by Soran et al. [44]. Thus, 0.01 mL of onion tail extract was added to 3.9 mL of DPPH-2,2 diphenyl-picryl-hydrazyl radical solution (0.0025 g/100 mL methanol). The obtained mixture was left in the dark for 10 min, after that the absorbance of the mixture was measured at 515 nm compared to a mixture obtained from 0.01 mL extract added to 3.9 mL methanol. The antioxidant capacity was determined using a calibration curve drawn for different concentrations of Trolox, obtained by successive dilution from the stock solution.

### 3.3. Multielemental Investigation of Onion Tail Samples by NAA

#### 3.3.1. Sample Handling and Condition of Measurements

Up to 80 mg samples were weighed on a digital microbalance and sealed in high-purity quartz ampoules (Suprasil AN, Heraeus). The samples were divided into 3 batches (3 × 6, +1). The quartz ampules were wrapped in aluminum foil and the set of 6 samples was encapsulated in an aluminum external container, irradiated all three in the same rotating, well-thermalized channel. The samples were irradiated for 12 h, with a set of monitor foils, Zr, Au 0.1%-Al (IRMM-530), and Fe, to get the neutron flux parameters, which are essential for the quantification in the concentration calculations by the *k_0_*-method. Neutron flux parameters have been calculated with the “Bare Triple-Monitor” approach. The thermal neutron flux density of rotating irradiation channels has been 1.86 × 1013 cm^−2^s^−1^, with f = 46.

The gamma rays emitted from the samples were counted within iron low-level counting chambers to reduce the room background. Two–three days after the irradiation, the short- or medium-lived radionuclides (like ^198^Au, ^139^Ba, ^82^Br, ^47^Ca, ^42^K, ^140^La and ^24^Na) were determined from a gamma spectrum that was measured for the first time for 900 s or longer. To improve the detection limit for several medium-lived radionuclides, a second measurement was made 1–3 days after the first measurement, and the third measurement another week later. When dominant short-lived isotopes had decayed, then the long-lived radionuclides could be determined (like ^60^Co, ^134^Cs, ^59^Fe, ^86^Rb, ^46^Sc, ^85^Sr, ^182^Ta and ^65^Zn).

#### 3.3.2. Instrumental Neutron Activation Analysis

Instrumental neutron activation analysis (INAA) of 19 onion tail samples was used to determine trace components of them with k0-NAA after a long irradiation with neutrons. The 19 samples were irradiated in four batches. Samples were irradiated in the Budapest Research Reactor, operated by the Centre for Energy Research (EK) (www.bnc.hu).

The gamma spectra of the samples were measured on two detectors: on the Canberra GC3618 HPGe p-type detector (36% relative efficiency, energy resolution 0.80 keV @ 59.5 keV, 1.75 keV @ 1332 keV, TRP preamp) and on the ORTEC HPGe p-type detector (55% relative efficiency, energy resolution 1.75 keV 1332 keV). Both detectors are operated in the 50–3300 keV energy range [47,48], and both are connected to a dual-input ORTEC DSPEC 502 digital gamma spectrometer and controlled by the ORTEC Maestro 7 software. The spectra with 2 × 16k channels were registered using the zero-dead time (ZDT) mode, in order to take into account exactly the different temporal dynamics of the isotopes of interest. The corrected and uncorrected halves of the spectra are stored in a single SPC file.

### 3.4. Data Analysis

The results are given as averages of three measurements ± SE (standard error). One-way analysis of variance (ANOVA) followed by Tukey’s test performed with Minitab 17 (Minitab Ltd., Coventry, UK) were used to assess the statistically considerable differences among groups (*p* < 0.05).

Microsoft Office Excel 2010 (Microsoft, Redmond, WA, USA) was used for the calculations and Origin 8 (OriginLab Corporation, Northampton, MA, USA) for drawing the graphs.

For spectrum evaluation, HyperLab 2013.1 software was used [49]. To identify the radioactive isotopes and element concentration calculations, the KayZero for Windows 3.06 program [50] was applied, which can determine thermal and epithermal neutron flux ratio (f), alpha, and F_c_ calculation factors. KayZero calculates concentrations using the k_0_-standardization method according to De Corte and Simonits [51].

## 4. Conclusions

The current study aims to follow the impact of contaminated soil with different concentrations of Cu, Cd, Mn, Ni, Zn and Pb on bioactive compounds, antioxidant capacity and elemental content from onions compared to the control.

The quantity of pigments (chlorophyll *a*, chlorophyll *b* and carotenoids) from the plants grown in the presence of heavy metals (Ni, Pb and Mn) decreased against the control indifferent of their concentration. On the other hand, in plants grown in the presence of Cu, Cd and Zn, the amount of pigments decreased in most cases. The total polyphenol content decreased in the plants grown in the presence of Ni, Pb and Mn and increased or decreased in the plants grown in the presence of Cu, Cd and Zn depending on the metal and its concentration. The antioxidant capacity decreased in onion tails grown in the presence of Ni, Pb and Mn and increased in the case of grown ones with Cu, Cd and Zn.

The k0-INAA method determined 14 chemical elements in the onion tails. Among the macronutrients, Ca and K were determined, while among the micronutrients, Fe, Na and Zn were determined. Co was released from the plant regardless of the treatment applied. Plant Br, K, Na, Rb and Zn concentration increased with applied Cu, Cd and Zn, the relative increase to control being over 297% for Na. With Mn, the plant significantly lost Br, Fe, K and Rb but strongly assimilated Zn, Au, Na and some Ca whose assimilation seems to be least affected by the additional presence of selected heavy metals in the soil. Increasing the Ni rate, as well as the Mn rate, diminished the plant Co, Fe, K, and Rb content, but leaf Zn and Au content increased. The variation in K and Rb content was always highly correlated with the variation in antioxidant capacity, regardless of the treatment applied. The additional presence of Ni, Pb and Mn in the soil induced a decrease of K and Rb and antioxidant capacity, while a simultaneous increase of K, Rb content and antioxidant capacity occurred with Cu, Cd and Zn.

## Figures and Tables

**Table 1:** Correlation of the elemental (Ca, K, Br, Co, Fe, Na, Rb, Zn, Au and Sc) concentration in *Allium cepa* L. (onion tails) with the concentration of heavy metals (Ni, Pb, Mn, Cu, Cd and Zn) applied to the soil. Blue marks negative correlations under −0.7; red marks positive correlations above 0.7; bold marks correlations under −0.9 and above 0.9.

Exp. Setup	Ca	K	Br	Co	Fe	Na	Rb	Zn	Au	Sc
Ni	−0.25	−0.35	−0.17	−0.65	−0.53	−0.66	−0.32	0.87	−0.32	-
Pb	**−0.96**	**−0.99**	−0.53	−0.68	−0.68	−0.27	**−0.96**	0.66	−0.24	−0.54
Mn	−0.15	−0.58	**−0.9**	0.21	−0.71	**0.95**	**−0.98**	**0.91**	0.85	-
Cu	−0.29	0.65	0.13	−0.75	−0.41	0.63	**0.96**	−0.07	-	-
Cd	−0.12	0.34	0.45	−0.78	0.54	0.51	0.78	0.54	-	-
Zn	0.30	0.34	0.05	−0.86	0.39	0.83	0.73	**0.96**	-	

**Table 2:** Correlation of elemental content in the plants for each experimental line. Blue marks negative correlations under −0.7; red marks positive correlations above 0.7; bold marks correlations under −0.9 and above 0.9.

Ni	Br	Ca	Co	Fe	K	Na	Rb	Zn	Au	Sc
Br	1									-
Ca	−0.41	1								-
Co	−0.28	−0.21	1							-
Fe	−0.10	−0.48	**0.96**	1						-
K	0.13	−0.74	0.81	**0.94**	1					-
Na	0.77	0.14	−0.05	−0.06	−0.03	1				-
Rb	−0.48	−0.33	**0.93**	**0.92**	0.80	−0.41	1			-
Zn	−0.57	0.19	−0.56	−0.58	−0.57	−0.78	−0.25	1		-
Au	**0.92**	−0.02	−0.37	−0.30	−0.16	**0.92**	−0.65	−0.56	1	-
Sc	-	-	-	-	-	-	-	-	-	-
**Pb**	**Br**	**Ca**	**Co**	**Fe**	**K**	**Na**	**Rb**	**Zn**	**Au**	**Sc**
Br	1									
Ca	0.35	1								
Co	0.10	0.56	1							
Fe	0.23	0.54	**0.99**	1						
K	0.46	**0.97**	0.69	0.69	1					
Na	−0.63	0.35	0.67	0.56	0.33	1				
Rb	0.40	**0.90**	0.85	0.85	**0.97**	0.45	1			
Zn	−0.18	−0.82	−0.02	0.03	−0.68	−0.13	−0.49	1		
Au	0.23	0.41	−0.52	−0.54	0.25	−0.35	−0.01	−0.83	1	
Sc	0.67	0.58	−0.25	−0.21	0.51	−0.50	0.29	−0.78	0.87	1
**Mn**	**Br**	**Ca**	**Co**	**Fe**	**K**	**Na**	**Rb**	**Zn**	**Au**	**Sc**
Br	1									-
Ca	−0.30	1								-
Co	0.11	−0.73	1							-
Fe	**0.91**	−0.51	0.51	1						-
K	0.83	−0.61	0.65	**0.99**	1					-
Na	−0.73	−0.43	0.47	−0.46	−0.31	1				-
Rb	**0.97**	−0.07	−0.01	0.85	0.74	−0.86	1			-
Zn	−0.75	−0.30	0.06	−0.67	−0.57	0.88	−0.88	1		-
Au	−0.73	−0.21	−0.11	−0.73	−0.64	0.78	−0.84	**0.98**	1	-
Sc	-	-	-	-	-	-	-	-	-	-
**Cu**	**Br**	**Ca**	**Co**	**Fe**	**K**	**Na**	**Rb**	**Zn**	**Au**	**Sc**
Br	1								-	-
Ca	−0.74	1							-	-
Co	−0.21	−0.12	1						-	-
Fe	0.73	−0.73	0.51	1					-	-
K	0.43	0.01	**−0.97**	−0.30	1				-	-
Na	0.65	−0.25	−0.88	−0.03	**0.96**	1			-	-
Rb	0.35	−0.32	−0.86	−0.30	0.82	0.82	1		-	-
Zn	**0.91**	−0.88	0.19	**0.93**	0.02	0.29	0.08	1	-	-
Au	-	-	-	-	-	-	-	-	-	-
Sc	-	-	-	-	-	-	-	-	-	-
**Cd**	**Br**	**Ca**	**Co**	**Fe**	**K**	**Na**	**Rb**	**Zn**	**Au**	**Sc**
Br	1								-	-
Ca	0.59	1							-	-
Co	−0.89	−0.45	1						-	-
Fe	0.29	0.54	−0.59	1					-	-
K	0.60	0.86	−0.70	0.88	1				-	-
Na	**0.97**	0.70	**−0.93**	0.52	0.78	1			-	-
Rb	0.66	0.50	**−0.90**	0.87	0.85	0.81	1		-	-
Zn	**0.98**	0.43	**−0.90**	0.21	0.48	0.92	0.63	1	-	-
Au	-	-	-	-	-	-	-	-	-	-
Sc	-	-	-	-	-	-	-	-	-	-
**Zn**	**Br**	**Ca**	**Co**	**Fe**	**K**	**Na**	**Rb**	**Zn**	**Au**	**Sc**
Br	1								-	-
Ca	−0.88	1							-	-
Co	−0.50	0.07	1						-	-
Fe	−0.84	**1.00**	−0.03	1					-	-
K	0.89	−0.57	−0.77	−0.50	1				-	-
Na	0.55	−0.14	**−1.00**	−0.04	0.80	1			-	-
Rb	0.71	−0.36	**−0.95**	−0.27	0.87	**0.97**	1		-	-
Zn	−0.04	0.28	−0.71	0.36	0.15	0.69	0.61	1	-	-
Au	-	-	-	-	-	-	-	-	-	-
Sc	-	-	-	-	-	-	-	-	-	-

**Table 3:** Correlation of elemental content (Ni, Br, Ca, Co, Fe, K, Na, Rb, Zn, Au and Sc) in *Allium cepa* L. with bioactive compounds (total polyphenols–TP, chlorophyll *a*–CHL*a*, chlorophyll *b*–CHL*b*, carotenoids-CARO) and antioxidant capacity (DPPH). Blue marks negative correlations under −0.7; red marks positive correlations above 0.7; bold marks correlations under −0.9 and above 0.9.

Ni	Br	Ca	Co	Fe	K	Na	Rb	Zn	Au	Sc
TP	0.60	−0.21	0.53	0.56	0.56	0.79	0.21	**−1.00**	0.59	-
DPPH	−0.07	−0.19	**0.97**	**0.93**	0.79	0.19	0.81	−0.74	−0.14	-
CHL*a*	−0.11	−0.28	**0.99**	**0.97**	0.85	0.09	0.87	−0.68	−0.22	-
CHL*b*	0.18	0.00	0.79	0.72	0.59	0.56	0.51	**−0.91**	0.23	-
CARO	−0.31	−0.30	**0.99**	**0.97**	0.85	−0.16	**0.97**	−0.48	−0.45	-
**Pb**	**Br**	**Ca**	**Co**	**Fe**	**K**	**Na**	**Rb**	**Zn**	**Au**	**Sc**
TP	0.79	0.38	0.64	0.74	0.57	−0.14	0.67	0.11	−0.33	0.18
DPPH	0.37	0.75	**0.95**	**0.96**	0.87	0.48	**0.96**	−0.24	−0.27	0.07
CHL*a*	−0.38	0.64	0.76	0.66	0.62	**0.94**	0.69	−0.38	−0.16	−0.21
CHL*b*	−0.57	−0.07	0.68	0.62	0.01	0.82	0.23	0.44	−0.82	−0.85
CARO	−0.24	0.78	0.73	0.64	0.75	0.86	0.78	−0.55	0.01	0.00
**Mn**	**Br**	**Ca**	**Co**	**Fe**	**K**	**Na**	**Rb**	**Zn**	**Au**	**Sc**
TP	0.51	0.32	−0.78	0.10	−0.05	−0.77	0.56	−0.39	−0.22	-
DPPH	**1.00**	−0.34	0.11	0.89	0.82	−0.70	**0.95**	−0.70	−0.67	-
CHL*a*	0.78	0.19	−0.53	0.44	0.29	**−0.90**	0.82	−0.65	−0.52	-
CHL*b*	0.68	−0.28	−0.35	0.38	0.30	−0.50	0.58	−0.19	−0.07	-
CARO	0.70	0.42	−0.60	0.37	0.20	**−0.98**	0.80	−0.76	−0.63	-
**Cu**	**Br**	**Ca**	**Co**	**Fe**	**K**	**Na**	**Rb**	**Zn**	**Au**	**Sc**
TP	−0.25	0.83	−0.43	−0.51	0.43	0.24	−0.09	−0.55	-	-
DPPH	−0.13	0.32	**−0.94**	−0.77	0.82	0.65	0.79	−0.49	-	-
CHL*a*	0.16	0.18	0.57	0.55	−0.40	−0.34	−0.81	0.27	-	-
CHL*b*	−0.05	0.46	0.43	0.27	−0.31	−0.33	−0.80	−0.02	-	-
CARO	0.16	0.00	0.75	0.67	−0.59	−0.49	−0.87	0.37	-	-
**Cd**	**Br**	**Ca**	**Co**	**Fe**	**K**	**Na**	**Rb**	**Zn**	**Au**	**Sc**
TP	0.59	0.23	−0.18	−0.56	−0.13	0.39	−0.22	0.60	-	-
DPPH	0.46	0.86	−0.58	**0.90**	**0.99**	0.67	0.79	0.33	-	-
CHL*a*	−0.66	−0.75	0.28	0.10	−0.39	−0.60	−0.06	−0.56	-	-
CHL*b*	−0.79	−0.88	0.52	−0.19	−0.64	−0.79	−0.36	−0.68	-	-
CARO	−0.70	−0.77	0.33	0.06	−0.42	−0.64	−0.12	−0.60	-	-
**Zn**	**Br**	**Ca**	**Co**	**Fe**	**K**	**Na**	**Rb**	**Zn**	**Au**	**Sc**
TP	−0.80	0.85	−0.05	0.86	−0.60	0.00	−0.18	0.64	-	-
DPPH	0.89	−0.57	−0.80	−0.50	**1.00**	0.83	**0.90**	0.21	-	-
CHL*a*	−0.51	0.11	0.62	0.05	−0.80	−0.62	−0.57	0.06	-	-
CHL*b*	−0.69	0.28	0.96	0.19	**−0.92**	**−0.97**	**−0.97**	−0.48	-	-
CARO	−0.54	0.12	0.70	0.06	−0.84	−0.69	−0.65	−0.03	-	-

## Data Availability

The original contributions presented in the study are included in the article; further inquiries can be directed to the corresponding authors.
